# Characterization of kilovoltage x‐ray image guidance system with a novel post‐processing algorithm on a new slip ring‐mounted radiotherapy system

**DOI:** 10.1002/acm2.14524

**Published:** 2024-09-11

**Authors:** Heling Zhu, Tingting Dong, Tingtian Pang, Qiu Guan, Jingru Yang, Feini Zhao, Bo Yang, Jie Qiu

**Affiliations:** ^1^ Department of Radiation Oncology, Peking Union Medical College Hospital Chinese Academy of Medical Sciences and Peking Union Medical College Beijing China; ^2^ Our United Corporation Xi'an Shanxi Province China

**Keywords:** kV‐CBCT, post‐processing algorithm, slip ring‐mounted Linac

## Abstract

**Purpose:**

This study evaluates the performance of a kilovoltage x‐ray image‐guidance system equipped with a novel post‐processing optimization algorithm on the newly introduced TAICHI linear accelerator (Linac).

**Methods:**

A comparative study involving image quality tests and radiation dose measurements was conducted across six scanning protocols of the kV‐cone beam computed tomography (CBCT) system on the TAICHI Linac. The performance assessment utilized the conventional Feldkamp–Davis–Kress (FDK) algorithm and a novel Non‐Local Means denoising and adaptive scattering correction (NLM‐ASC) algorithm. Image quality metrics, including spatial resolution, contrast‐to‐noise ratio (CNR), and signal‐to‐noise ratio (SNR), were evaluated using a Catphan 604 phantom. Radiation doses for low‐dose and standard protocols were measured using a computed tomography dose index (CTDI) phantom, with comparative measurements from the Halcyon Linac's iterative CBCT (iCBCT).

**Results:**

The NLM‐ASC algorithm significantly improved image quality, achieving a 300%–1000% increase in CNR and SNR over the FDK‐only images and it also showed a 100%–200% improvement over the iCBCT images from Halcyon's head protocol. The optimized low‐dose protocols yielded higher image quality than the standard FDK protocols, indicating potential for reduced radiation exposure. Clinical implementation confirmed the TAICHI system's utility for precise and adaptive radiotherapy.

**Conclusion:**

The kV‐IGRT system on the TAICHI Linac, with its novel post‐processing algorithm, demonstrated superior image quality suitable for routine clinical use, effectively reducing image noise without compromising other quality metrics.

## 1 INTRODUCTION

In the rapidly developing field of radiotherapy, precision and accuracy in tumor targeting and treatment delivery are crucial. Cone beam computed tomography (CBCT) has become an essential tool in this effort, providing three‐dimensional imaging integral to effective radiotherapy execution. CBCT's integration into radiotherapy workflows has been transformative, offering real‐time imaging capabilities for precise tumor localization and adaptive treatment strategies.^1^, ^2^, ^3^ Recently, image‐guided adaptive radiation therapy (ART) has shown promise in improving treatment outcomes, with multiple studies demonstrating its potential benefits in enhancing target coverage while protecting organs at risk (OARs).^4^, ^5^, ^6^ High‐quality and accurate images are key factors for adaptive treatment. However, the full potential of CBCT has historically been limited by challenges related to image quality, such as shape distortion, magnification, noise, scattering artifacts, and the balance between image quality and radiation dose.

To address these issues, recent research efforts have aimed to improve CBCT image quality. Jin et al.^7^ utilized a beam‐block approach to measure scattering and applied this correction to CBCT images. Lei et al.^8^ developed a random forest‐based method to enhance CBCT image quality. Yang et al.^9^ implemented a cycleGAN‐based synthetic CT algorithm to improve CBCT imaging. Despite these advancements, most optimization algorithms have not been widely applied to online CBCT scans. One the one hand, deep learning‐based optimization algorithms require significant generalization capabilities when used online; one the other hand, complex algorithms and models may result in excessive optimization and reconstruction times and even GPU dependence, which are generally impractical for online treatments. Varian's iterative CBCT (iCBCT) is a notable exception, enabling efficient and robust IGRT in an online setting.^10^, ^11^

A novel TAICHI Linac (OUR United Corp., Xi'an, China) was put into use at Peking Union Medical College Hospital (PUMCH) in July of 2023. It features an enclosed, slip ring‐mounted gantry, a kilovoltage imaging system, and a 6 MV flattening filter‐free (FFF) beam. The gantry can start a full circle rotation at any angle, facilitated by a slip ring, serving as an electrical and signal connection between the stationary and rotating parts. The kV CBCT scanning speed on the TAICHI Linac is 9 degrees per second, which is faster than the 6 degrees per second of conventional C‐arm Linacs and is comparable to some advanced imaging function like HyperSight on TrueBeam Linac. One advantage of the TAICHI Linac's CBCT acquisition is its ability to start from any angle, reducing the gantry positioning preparation time. Additionally, the kV CBCT system on the TAICHI Linac is equipped with an innovative post‐processing optimization algorithm that significantly improves online CBCT image quality, which is detailed in this article.

In this study, six commonly used scanning protocols on the TAICHI system have been investigated. We evaluated the overall performance of the CBCT image with a novel online post‐processing optimization algorithm on the TAICHI system and compared it with the iCBCT on the Halcyon system.

## 2 MATERIALS AND METHODS

### 2.1 Overview of the kilovoltage (kV) volumetric CBCT system on TAICHI Linac

The TAICHI Linac device is equipped with a kilovoltage (kV) imaging system, comprising a kV x‐ray source, a high‐voltage generator, a field adjustment device, and a kV x‐ray detector. These components are all mounted on the machine's annular gantry, positioned coaxially and coplanar with the gantry rotation system. The source‐to‐imager distance (SID) is set at 184 cm, and the source‐to‐axis distance (SAD) measures 115 cm.

**FIGURE 1:**
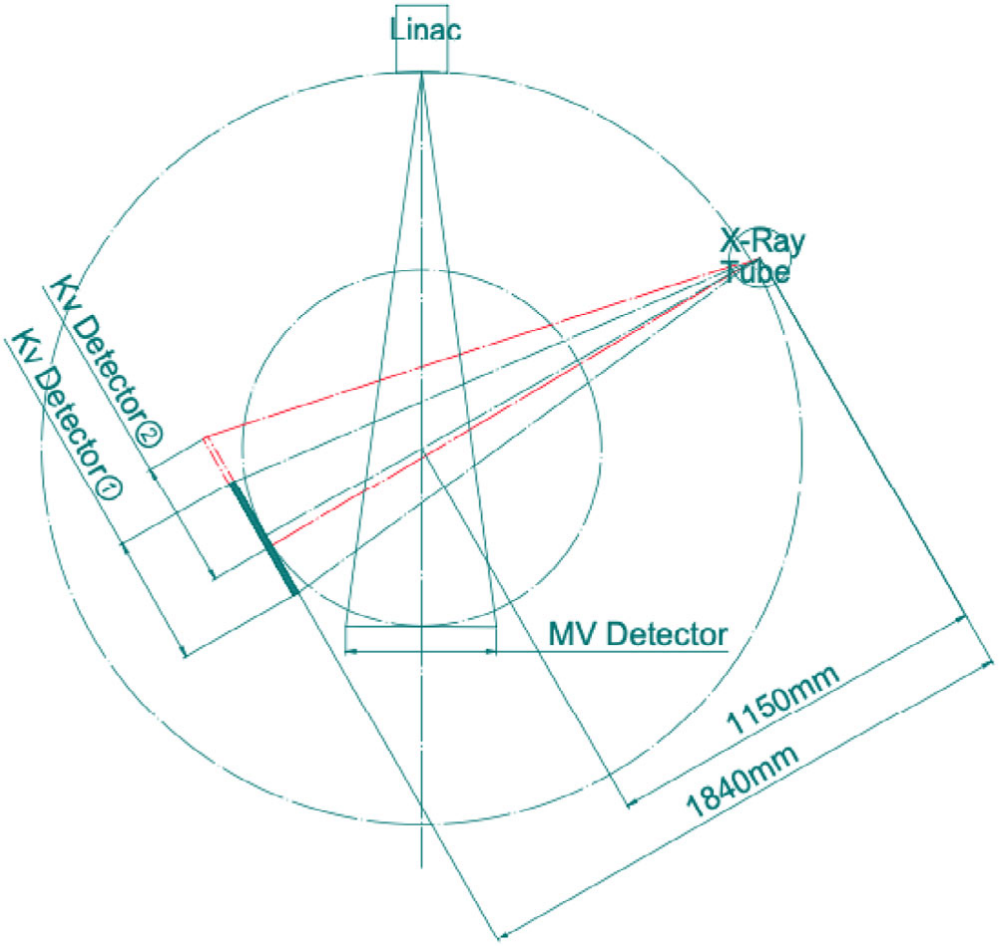
The configuration of TAICHI kV and MV beamline system.

Figure 1 illustrates the configuration of the kV imaging system and MV beamline on the gantry. The imaging area size of the kV x‐ray detector is 40.96 cm × 40.96 cm. A matrix size of 512 × 512 and a slice thickness of 2 mm are pre‐programmed for the reconstruction of all protocols. Relative to the kV source, the detector can be positioned in two ways: The first position aligns the detector center directly opposite the x‐ray tube, enabling an axisymmetric scan (labeled as kV Detector ① in Figure 1). The second position involves laterally offsetting the detector by 16 cm for a non‐axisymmetric scan (labeled as kV Detector ② in Figure 1). The axisymmetric scan is commonly used for clinical head scans with a field of view (FOV) of 25 cm in diameter, and the maximum coverage in the head‐to‐foot direction can reach 25 cm. The non‐axisymmetric scan mode is often employed for clinical body scans, offering an FOV of 44.5 cm in diameter, with the maximum length in the head‐to‐foot direction also extending up to 25 cm.

A kV CBCT imaging system that offers nine scanning protocols is currently in clinical use, with the details of these protocols presented in Table 1. In addition, we have compared these results with those of three analogous scanning protocols—Head, Thorax, and Pelvic Standard—on the Halcyon system at our center. Table 2 outlines the technical specifications of the three Halcyon CBCT protocols employed for comparison. Notably, the iCBCT algorithm on the Halcyon system supports only the head and pelvic scanning protocols. Therefore, the Thorax standard protocol from the Halcyon system was included only for the comparison of CTDI results in this study.

**TABLE 1:** The kV CBCT scanning protocols provided by TAICHI system.

Modes on TAICHI	Energy (kV)	Default (mAs)	Number of proj.	mA/ms per proj.	Scan time (s)	Recon. FOV (mm)
Head standard (SD)	100	96	300	20/16	23	250
Head high quality (HQ)	100	154	300	32/16	23	250
Thorax low dose (LD)	120	212	530	40/10	40	445
Thorax standard (SD)	120	339	530	64/10	40	445
Thorax large	120	678	530	64/20	40	445
Pelvic low dose (LD)	120	678	530	64/20	40	445
Pelvic standard (SD)	120	1085	530	64/32	40	445
Pelvic large	120	1325	530	100/25	40	445
Pelvic large+	120	1656	530	125/25	40	445

**TABLE 2:** Three scanning protocols used for comparison on Halcyon system.

Modes on Halcyon	Energy (kV)	Default (mAs)	Number of proj.	mA/ms per proj.	Scan time (s)	Recon. FOV (mm)
Head standard	100	139	463	30/10	16.6	281
Thorax standard	125	301	859	35/10	30.8	491
Pelvic standard	125	1074	895	80/15	36.7	491

All CBCT modes in the TAICHI system support image reconstruction with the conventional Feldkamp–Davis–Kress (FDK)‐based algorithm. Furthermore, we have developed a post‐processing optimization algorithm that employs non‐local means (NLM) denoising and adaptive scattering correction (NLM‐ASC) to enhance the image quality of CBCT images. More details about this algorithm will be provided in the following section.

### 2.2 Algorithm of CBCT optimization

The CBCT optimization algorithm encompasses two critical processes: noise reduction and scattering correction. The noise reduction component utilizes the NLM algorithm, inspired by the approach detailed in Yan et al.^12^ This method, as described by Equation 1, reduces noise by adaptively adjusting the weights assigned to neighboring pixels, averaging those that are similar to a greater degree.

(1) $$\begin{array}{c} f_{CBCT}\left(x\right)=\frac{1}{Z}\left[\sum_{y}\left(w_{y}\cdot f_{CBCT}^{\mathrm{Ori}}\left(y\right)\right)+w^{\mathrm{Ori}}\cdot f_{CBCT}^{\mathrm{Ori}}\left(x\right)\right] \end{array}$$

where $f_{CBCT}^{\mathrm{Ori}}\left(x\right)$ denotes the intensity value of the original CBCT image at voxel x; $f_{CBCT}\left(x\right)$ represents the value at the same voxel in the noise‐reduced image. The weight $w^{Ori}$ is determined based on empirical data, and $w_{y}$ is calculated by assessing the similarity between voxel x and every voxel y within its neighborhood V. The larger V is, the better the denoising effect is, but the processing time is longer. Summarizing our experience of parameter adjustment, we took a neighborhood voxel range of 11 pixels in each direction. This similarity is quantified by:

(2) $$w_{y}=\;\exp\left\{-\frac{1}{h^{2}}\cdot {∥V^{\mathrm{Ori}}\left(x\right)-\;V^{\mathrm{Ori}}\left(y\right)∥}_{2}^{2}\right\}$$

where *h* is an adjustable parameter tailored to the noise level within the current processing block $V^{\mathrm{Ori}}\left(y\right)$; lower noise levels necessitate a smaller *h*, which consequently reduces the weight $w_{y}$, and the inverse is true for higher noise levels. And in Equation 1, the term $Z\;=\sum_{y}w_{y}\;+w^{\mathrm{Ori}}$ serves as a normalization factor to ensure the sum of the weights equals one, thereby preserving the relative intensities of the image.

After the above processing, CBCT images can be significantly improved in noise reduction while retaining the clarity of image edges. In order to further reduce scattering artifacts in CBCT images, we adopted the scattering optimization method proposed in Zhen et al.^13^ This involves using the planning CT images acquired after image registration to establish specific thresholds. Scattering correction is subsequently applied according to these predefined thresholds in the CBCT images. This step significantly enhances image uniformity and reduces artifacts, leading to clearer and more accurate diagnostic images.

The algorithm was developed using C language and integrated into the imaging system software of TAICHI system for online processing. During treatment, this algorithm performs post‐processing immediately after image acquisition and reconstruction. The processing time is around 2 s, and then, it is displayed on the user interface for registration review.

### 2.3 Phantoms used for kV image quality test

#### 2.3.1 Image quality measurement with the Catphan 604 phantom

**FIGURE 2:**
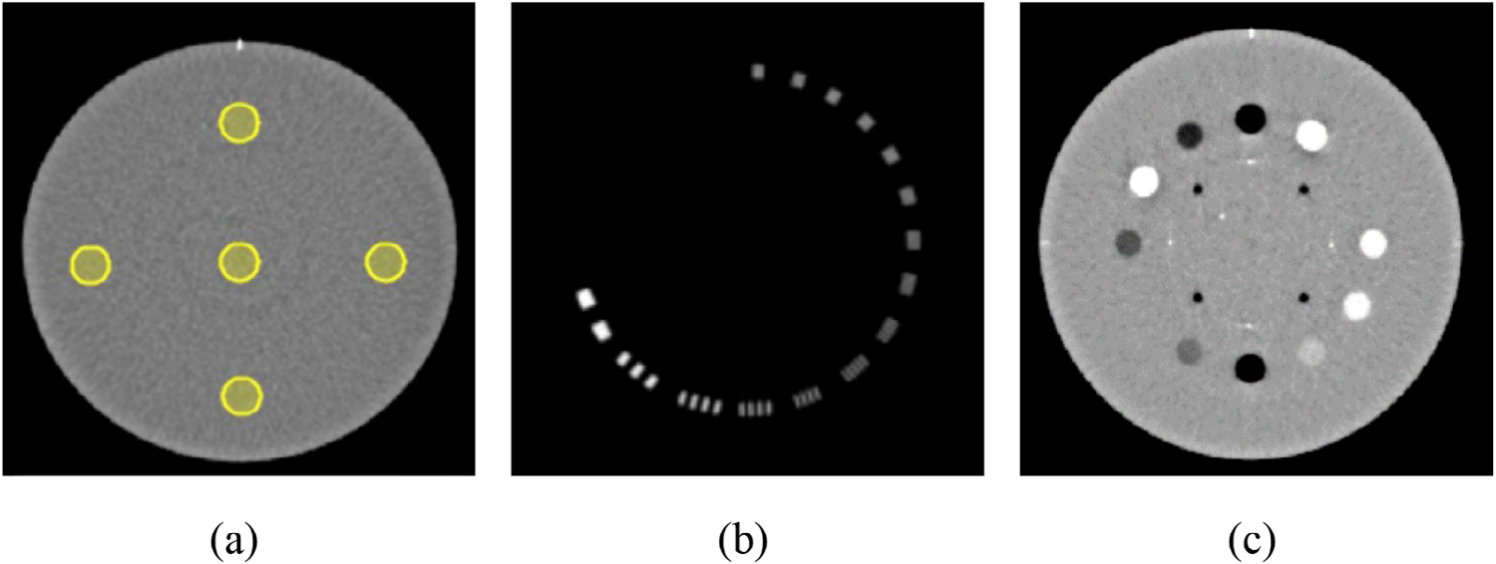
Slices of three modules in Catphan 604 phantom. (a) CTP729, (b) CTP730, CTP732.

The Catphan 604 phantom (The Phantom Laboratory, Salem, New York, USA) was employed to evaluate the image quality of kV‐CBCT, focusing on the metrics that will be discussed in the subsequent section. As shown in Figure 2, there are three modules in the phantom: CTP729, CTP730, and CTP732. The CTP729 module is utilized to assess the CT number uniformity and image noise. The CTP730 module focuses on evaluating the high‐contrast resolution of the image. The CTP732 module is dedicated to examining the CT number accuracy, slice thickness accuracy, and spatial linearity of the image. Further details can be found in the Catphan 604 manual.^14^

#### 2.3.2 kV volumetric imaging dose measurement

**FIGURE 3:**
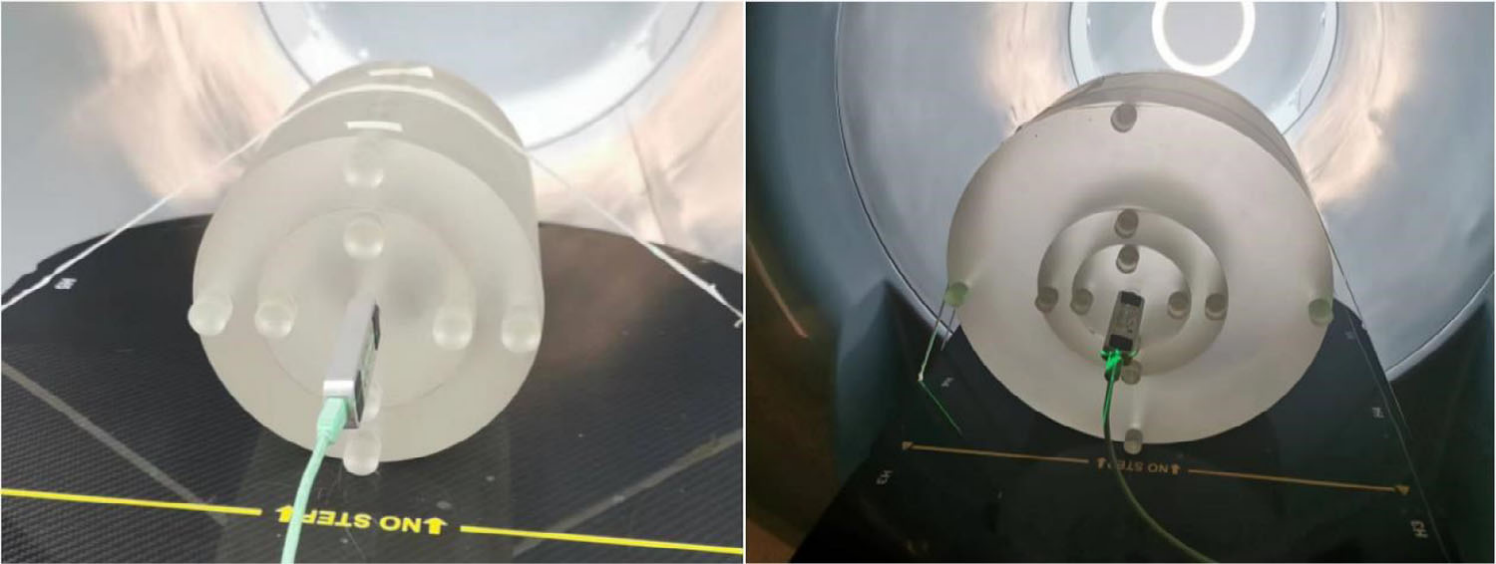
Schematic diagram of CTDI phantom measurement. Left: the 16 cm head phantom; right: the 32 cm body phantom.

For measuring the kV volumetric imaging dose in this study, the computed tomography dose index (CTDI) phantom and the Unfors CT detector (Unfors RaySafe AB) as depicted in Figure 3, was utilized. The 16 cm phantom is specifically employed for the Head protocols, while the 32 cm one is designated for body protocols including thorax and pelvic sites. For the dose measurement, the detector is inserted into one of the five cavities present in the CTDI phantom, while the remaining cavities are plugged with acrylic cylinders to ensure accuracy in the readings obtained.

### 2.4 Evaluation metrics of image quality

#### 2.4.1 High‐contrast (spatial) resolution

In the domain of medical imaging, the modular transfer function (MTF) stands as the quintessential metric for assessing high‐contrast resolution. In this study, we measured the spatial frequencies where MTF is 50% of its low‐frequency value (MTF50). The DoseLab software (Varian Medical Systems) was used to calculate the MTF. First of all, the modulation for each spatial frequency ROI in the CTP730 module of the Catphan 604 phantom was calculated by:

(3) $$\mathrm{Modulation}\;=\;\frac{S90-S10}{S90+S10}$$

where S90 is the 90^th^ percentile signal level (HU values) in the ROI, and S10 is the 10^th^ percentile signal level in the ROI. Using the 90^th^ and 10^th^ percentile values allows for a more forgiving placement of the ROI and does not allow outlier pixel values ​​to drastically change the results. Then, the modulations of ROIs were normalized to their highest value and the MTF was plotted as the modulation versus spatial frequency in line pairs per centimeter (lp/cm) of each ROI.

#### 2.4.2 Contrast‐to‐noise ratio (CNR)

Contrast‐to‐noise‐ ratio (CNR) values were derived from four distinct inserts [air, Teflon, low‐density polyethylene (LDPE), and acrylic] located in the CTP732 module of the Catphan 604. The process involves delineating the average computed tomography (CT) numbers within regions of interest (ROIs) inside the inserts as well as in an adjacent area. The diameter of each ROI in this measurement was 8.5 mm. These values are then utilized to calculate CNR as depicted in Equation 4. Noise is determined based on the standard deviation of the background values.

(4) $$CNR_{i}=\;\frac{\left|mean\;HU\;of\;insert-mean\;HU\;of\;background\right|}{noise}$$

#### 2.4.3 Signal‐to‐noise ratio (SNR)

The SNR is calculated as the mean image signal within a specified region divided by the noise in that region. For this measurement, the image uniformity module (CTP729), made from a uniform material, is utilized. A 10 cm × 10 cm area at the center of the CTP729 module in the Catphan 604 phantom is selected for analysis. Within this area, both the mean and the standard deviation of the CT numbers are measured. The SNR is then determined using Equation 5, where the noise is defined as the standard deviation of the CT numbers in the selected area.

(5) $$\mathrm{SNR}\;=\;\frac{\mathrm{mean}\;\mathrm{HU}\;\mathrm{of}\;\mathrm{selected}\;\mathrm{area}}{\mathrm{noise}}\times 100\%$$

#### 2.4.4 Slice thickness accuracy

The approach to measure the slice thickness involves using the CTP732 module. This process includes measuring the full width at half maximum (FWHM) of a projection from a 23° wire ramps. The measured slice thickness, denoted as Zmm, is then calculated according to Equation 6.

(6) $$Zmm\;=\;\mathrm{FWHM}\;\times \;tan23^{\circ }$$

### 2.5 Dosimetry measurement

The radiation dose of images serves as a crucial indicator of the performance of radiotherapy imaging systems. In this study, the measurement of imaging dose in this study refers to the American Association of Physicists in Medicine (AAPM) Task Group 75 report and related literature.^15^, ^16^ The parameter used to measure kV imaging dose was weighted CTDI (CTDIw), which can be directly compared with the vendor‐provided data on Halcyon. The formula for calculating the kV imaging dose is encapsulated in Equation 7, which considers $CTDI_{100}\left(Periphery\right)$ as the average dose measured at the phantom's periphery and $CTDI_{100}\left(Center\right)$ as the dose measured at the center of the phantom.

(7) $$CTDIw\;=\;\frac{2}{3}CTDI_{100}\left(Periphery\right)+\frac{1}{3}CTDI_{100}\left(Center\right)$$

### 2.6 Clinical implementation

The optimization algorithm was further applied to real patient images to demonstrate the feasibility of clinical implementation of this system. CBCT image sets from the head, thorax, and pelvic regions of three patients, who had previously undergone treatment on the TAICHI Linac, were randomly chosen. For the purposes of this study, these images were used for qualitative evaluation only in this study.

## 3 RESULTS

### 3.1 Measured image quality from Catphan 604 phantom

A total of 12 CBCT image sets were acquired using the six most commonly employed scanning protocols on the TAICHI system. These images were processed with the Feldkamp–Davis–Kress (FDK) reconstruction algorithm both before and after the application of the NLM denoising and adaptive scattering correction (NLM‐ASC) algorithm. Within all tables and figures presented in this article, results obtained solely with the FDK reconstruction algorithm are denoted as “FDK‐only,” while those processed with the NLM‐ASC optimization are labeled as “NLM‐ASC.” Furthermore, for comparative analysis, two sets of Halcyon iCBCT images scanned under Head and Pelvic standard protocols were also collected and evaluated.

#### 3.1.1 High‐contrast resolution evaluation

**FIGURE 4:**
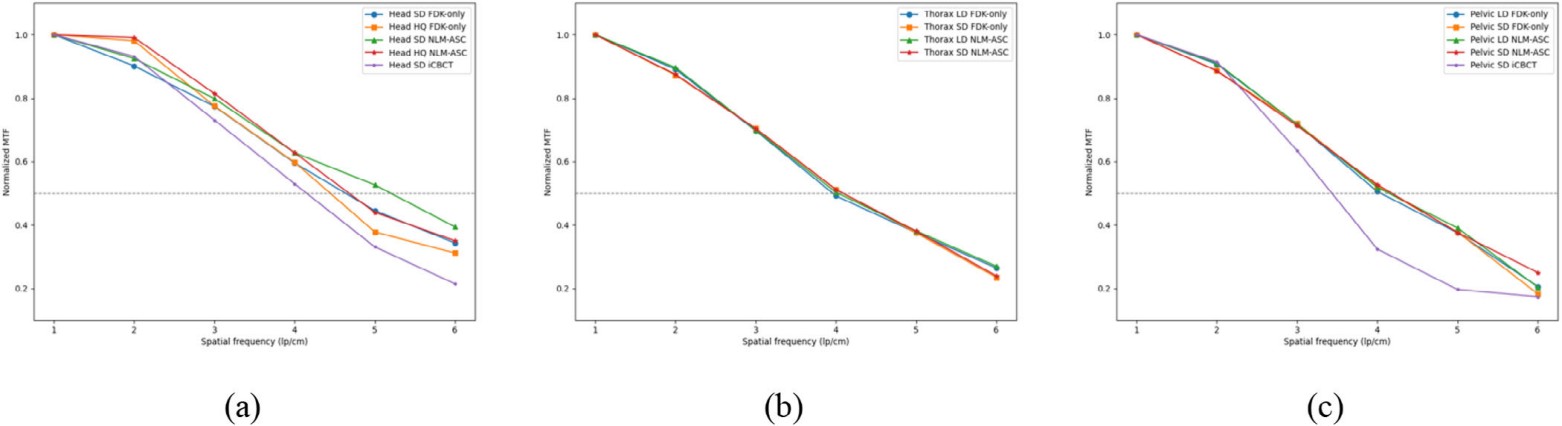
The measured MTF curves of (a) head, (b) thorax, and (c) pelvic scanning protocols.

Table 3 delineates the high‐contrast resolution values obtained through 50% of MTF including all 12 CBCT image sets from the TAICHI Linac and two iCBCT‐based images from the Halcyon Linac. For both head and pelvic protocols, the images from TAICHI Linac demonstrated slightly better results than those from the Halcyon's iCBCT‐based images. The difference between FDK‐only and NLM‐ASC optimized images was minor, since the NLM‐ASC algorithm was not designed to increase spatial resolution. For better comparison, Figure 4 exhibits the measured MTF curves of head, thorax, and pelvic scans respectively.

**TABLE 3:** High‐contrast resolution presented as 50% of MTF (MTF50) of CBCT image sets on TAICHI Linac and iCBCT‐based image sets on Halcyon Linac.

	FDK‐only		NLM‐ASC		Halcyon iCBCT
Metrics	LD (SD for Head)	SD (HQ for Head)	LD (SD for Head)	SD (HQ for Head)	SD
Head	4.6 lp/cm	4.4 lp/cm	5.2 lp/cm	4.7 lp/cm	4.1 lp/cm
Thorax	4.0 lp/cm	4.1 lp/cm	4.0 lp/cm	4.1 lp/cm	∕
Pelvic	4.0 lp/cm	4.2 lp/cm	4.1 lp/cm	4.2 lp/cm	3.4 lp/cm

The iCBCT algorithm on Halcyon is not implemented on the Thorax protocol.

#### 3.1.2 CNR and SNR enhancement through optimization

Tables 4, 5, 6 show the values of CNR and SNR for different image sets, illustrating the positive correlation between increased dose and the improvement in both CNR and SNR across all images. Notably, the CNR and SNR in NLM‐ASC optimized images increased by approximately 300%−1000% relative to FDK‐only images under identical scanning parameters. This enhancement was evident across all protocols, where the quality of NLM‐ASC‐optimized low‐dose (standard for head protocols) images surpassed that of FDK‐only standard (high‐quality for head protocols) images. This significant improvement underscores the optimization algorithm's potential in reducing patient radiation dose without compromising image quality.

**TABLE 4:** CNR and SNR values of FDK‐only, NLM‐ASC‐based and iCBCT‐based images for head protocols.

		FDK‐only		NLM‐ASC		Halcyon iCBCT
	Metrics	Standard	High quality	Standard	High quality	Standard
CNR	Air	15.87	21.48	113.54	139.98	52.39
	Teflon	14.83	19.09	85.51	84.18	42.60
	LDPE	2.72	3.68	18.39	21.66	5.35
	Acrylic	1.15	1.64	6.78	8.14	4.71
SNR		24.52	30.75	119.39	147.40	33.15

**TABLE 5:** CNR and SNR values of FDK‐only and NLM‐ASC‐based images for thorax protocols.

		FDK‐only		NLM‐ASC	
	Metrics	Low dose	Standard	Low dose	Standard
CNR	Air	28.89	34.53	150.42	184.28
	Teflon	20.25	27.81	80.53	158.71
	LDPE	3.47	4.93	16.21	27.21
	Acrylic	1.79	1.95	9.73	9.92
SNR		37.68	48.57	162.61	240.19

The iCBCT algorithm on Halcyon is not implemented on the thorax protocol.

**TABLE 6:** CNR and SNR values of FDK‐only, NLM‐ASC‐based and iCBCT‐based images for pelvic protocols.

		FDK‐only		NLM‐ASC		Halcyon iCBCT
	Metrics	Low dose	Standard	Low dose	Standard	Standard
CNR	Air	54.16	62.19	272.08	416.6	801.53
	Teflon	43.07	55.17	186.67	348.64	165.08
	LDPE	7.15	10.29	57.37	112.43	84.49
	Acrylic	3.01	3.79	27.71	37.59	72.18
SNR		68.95	87.73	280.64	382.11	386.35

Furthermore, for head protocols, NLM‐ASC‐optimized images on the TAICHI Linac outperformed iCBCT‐based images from the Halcyon system in terms of CNR and SNR. Meanwhile, for pelvic protocols, the performance metrics were comparable between the TAICHI and Halcyon Linacs.

#### 3.1.3 Assessment of reconstruction slice thickness

**FIGURE 5:**
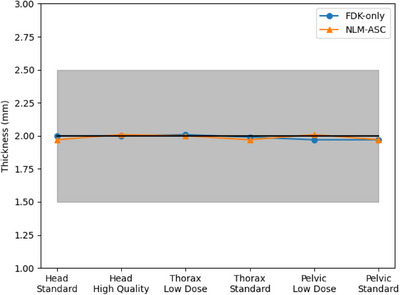
The consistency of slice thickness across methodologies is illustrated. The blue line, marked with circles, denotes the measurements using the FDK‐only algorithm. The orange line, distinguished by triangle markers, represents the results from the NLM‐ASC optimization algorithm. The expected range of 2 mm ± 0.5 mm tolerance is depicted by the black line and the surrounding gray shaded area, indicating that all measured values fall within this acceptable range.

The reconstruction slice thickness for all images was 2 mm. Measurements of the slice thickness, as visualized in Figure 5, were within the ± 0.5 mm tolerance range. This analysis revealed no significant variation in slice thickness across different protocols or between the two optimization algorithms utilized.

### 3.2 Radiation dose comparison

**FIGURE 6:**
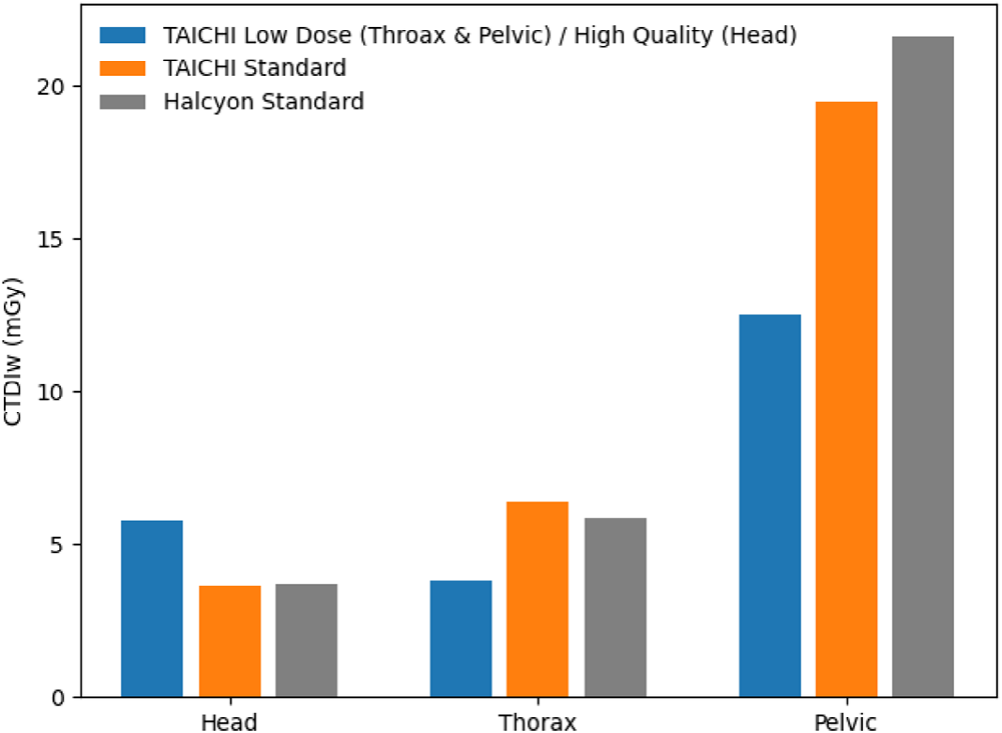
Measured imaging dose with CTDI phantom. The blue bar represents the measured CTDIw of head high‐quality, thorax low‐dose, and pelvic low‐dose protocols on TAICHI from left to right respectively; the orange bar represents the measured CTDIw of standard protocol on TAICHI; the gray bar represents the vendor‐provided CTDIw of standard protocol on Halcyon.

The CTDIw for the six scanning protocols on the TAICHI Linac is presented alongside the vendor‐provided data of “Standard” protocols on Halcyon Linac are shown in Figure 6. A comparative analysis of the standard scanning protocols for identical anatomical sites reveals nuanced differences in scanning doses. Specifically, for the head and pelvic standard protocols, the radiation doses associated with the Halcyon Linac are slightly higher than those of the TAICHI Linac. Conversely, for the thorax protocol, the dose from the Halcyon system is slightly lower.

### 3.3 Clinical image comparison

**FIGURE 7:**
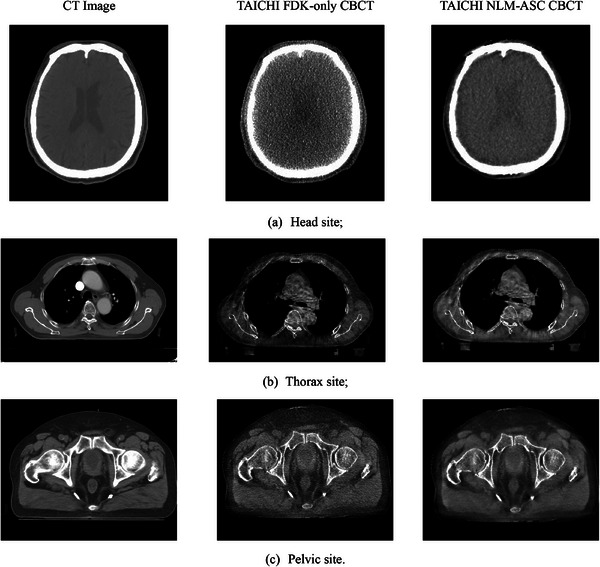
The acquired patient images from both CT simulator and TAICHI Linac of head, thorax, and pelvic sites. Left column: CT images; middle column: TAICHI CBCT with FDK‐only reconstruction algorithm; TAICHI CBCT after NLM‐ASC optimization algorithm. The scanning protocols are (a) head standard, (b) thorax standard, and (c) pelvic standard, respectively.

The optimization algorithm was subsequently applied in the clinical setting of the TAICHI system. The entire process was smooth and efficient, leading to a noticeable improvement in the visual quality of the reconstructed patient images after optimization. Figure 7 displays a series of kV CBCT images from the TAICHI system and the patient's original CT images acquired on a multi‐slice CT simulator for the head, thorax, and pelvic sites. While some artifacts persist in the CBCT images even after NLM‐ASC optimization, the image quality is notably closer to that of conventional CT images and significantly surpasses that of CBCT images reconstructed with the FDK‐only algorithm, as can be discerned even visually.

**FIGURE 8:**
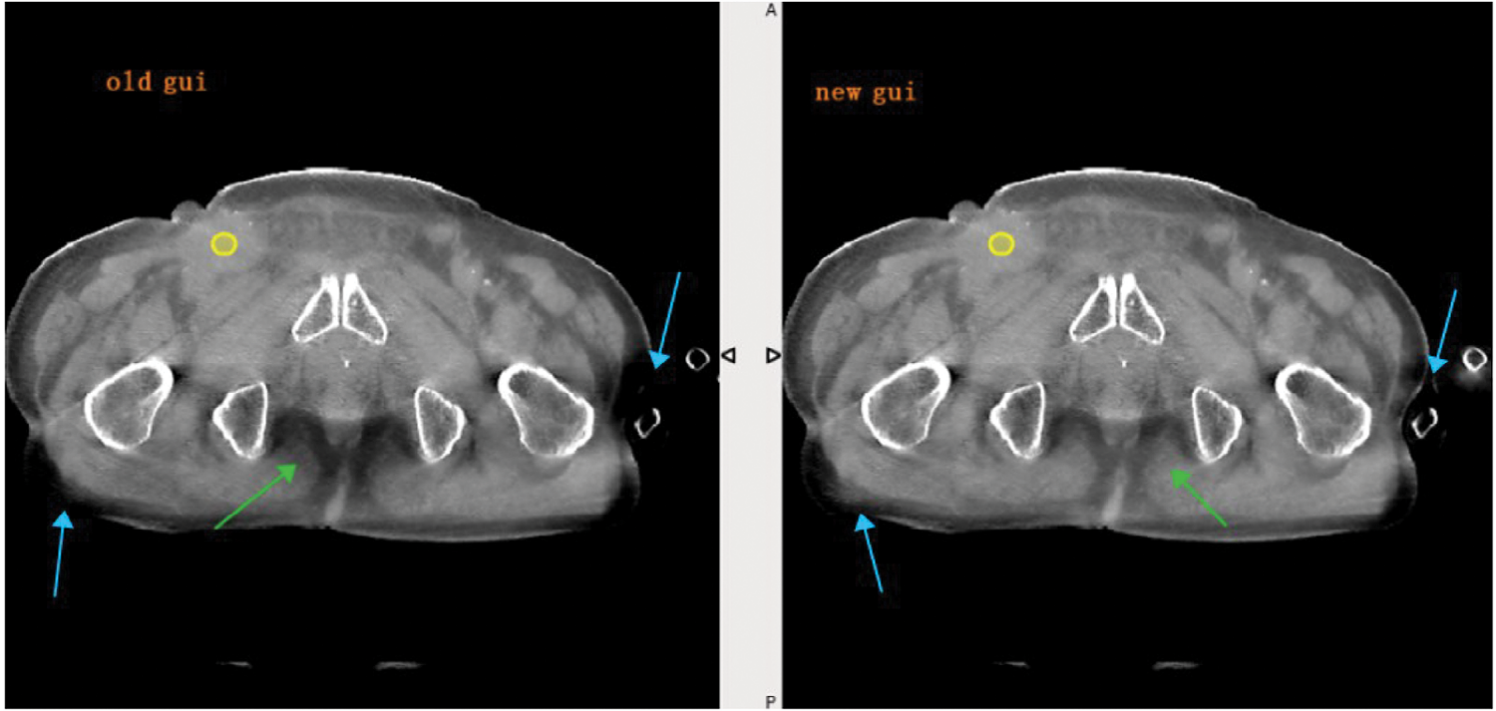
An example of a pelvic image with uneven dark artifact. Left: before NLM‐ASC optimization; Right: after NLM‐ASC optimization.

Figure 8 presents a specific example of a pelvic image. In this instance, three areas highlighted by arrows exhibit uneven dark regions that demonstrate considerable amelioration after the optimization process. This visual comparison proved the optimization algorithm's efficacy in enhancing image quality, offering clinicians a more refined tool for patient evaluation and treatment planning.

## 4 DISCUSSION

This study represents the first comprehensive evaluation of the kilovoltage x‐ray image guidance system equipped with the novel post‐processing optimization algorithm (NLM‐ASC) on the TAICHI Linac. To our knowledge, there is a lack of existing literature describing or evaluating the performance of this new kV CBCT system. Our results demonstrate significant enhancements in image quality metrics, showing the system's capacity to improve image‐guided radiation therapy (IGRT) with a streamlined and efficient process.

Utilizing the Catphan 604 phantom, we evaluated the image quality under various protocols on the TAICHI Linac. The performed tests and QA criteria used in this work were principally adapted from AAPM guidelines.^17^, ^18^ High‐contrast resolution (also known as spatial resolution) is a key parameter used to evaluate imaging systems. It is characterized by the imaging system's ability to distinguish two very small objects placed closely together. The results of spatial resolution presented as MTF50 from TAICHI Linac ranged between 4 and 5 lp/cm, which is similar to previously reports on the order of 3−5 lp/cm for kV CBCT.^19^, ^20^, ^21^ The head protocol displayed superior resolution compared to body site protocols, and for both head and pelvic protocols, spatial resolutions of images from TAICHI Linac show slightly better results than those from the Halcyon's iCBCT‐based images.

Image noise, representing random fluctuations and systematic uncertainties in imaging facility, critically impacts CBCT image quality by degrading spatial resolution. Our analysis of noise metrics, including CNR and SNR, revealed that the TAICHI system's NLM‐ASC optimization significantly outperforms traditional FDK reconstruction methods. This advancement is crucial for the precise delineation of tumor boundaries and organs at risk. Additionally, as evidenced by the markedly higher CNR and SNR values in low‐dose CBCT images compared to standard FDK‐only images, this confirms the optimization algorithm's role in reducing imaging exposure doses during clinical treatment. When compared to Halcyon's advanced online iCBCT technology, the TAICHI CBCT with NLM‐ASC optimization exhibits superior performance in head protocol scans in terms of CNR and SNR values, with mixed results in pelvic protocol evaluations.

Furthermore, this study utilized a CTDI phantom to measure the imaging dose across six scanning protocols on the TAICHI system. The imaging dose for pelvic and thorax sites under the low‐dose protocol was approximately 40% lower than the standard protocol, while for head scans, the high‐quality protocol's imaging dose was about 60% higher than the standard protocol. Compared to the vendor‐provided data of head and pelvic standard protocols on Halcyon Linac, TAICHI's imaging doses were slightly lower. Moreover, Islam et al.^16^ reported the dose metrics for the Elekta XVI kV‐CBCT imaging system, revealing that under test conditions, specifically a FOV of 26 cm by 26 cm, a tube voltage of 120 kVp, and a tube current of 660 mAs, the recorded center and surface doses on a body CTDI phantom were 16 and 23 mGy, respectively. In contrast, our investigation into the TAICHI kV‐CBCT system's performance under the pelvic low‐dose protocol, maintaining the same tube voltage (120 kVp) and a comparable tube current (678 mAs), yielded a CTDIw of 12.5 mGy. Notably, this was achieved over a larger maximum body scanning FOV of 44.5 cm × 25 cm.

The practical utility of the TAICHI system's NLM‐ASC algorithm is further validated through its clinical implementation, as evidenced by patient images. The optimization algorithm not only enhances the visual quality of CBCT images, but also reduces artifacts significantly. This improvement is crucial for adaptive radiotherapy strategies, requiring accurate anatomical details for daily treatment adaptation. Additionally, the TAICHI system's design, featuring a slip ring‐mounted gantry for continuous rotation, supports rapid and flexible imaging workflows, enhancing patient comfort and reducing the likelihood of motion artifacts.

## 5 CONCLUSION

The performance of the kV‐IGRT system on the TAICHI Linac presented here demonstrated that the integration of a post‐processing optimization algorithm, namely NLM‐ASC, markedly improved the image quality of kV CBCT system. This enhancement resulted in images with significantly reduced noise and scattering artifacts, offering clearer visualization for clinical use. When these images were compared with those generated by the iCBCT system on Halcyon, the CBCT images from TAICHI showed comparable performance across all evaluated image quality metrics, particularly with better results in terms of CNR and SNR for head protocols. Moreover, the NLM‐ASC optimization algorithm enables the use of lower radiation dose protocols without compromising image quality, contrasting with the conventional FDK only reconstruction approach. In conclusion, the kV‐IGRT system on the TAICHI Linac showed advantages in image quality, which can provide great help for everyday image‐guided radiotherapy and future adaptive radiotherapy. In the meantime, the test procedures and results designed in this study can provide a reference for future imaging system testing for users of TAICHI Linac.

## AUTHOR CONTRIBUTIONS

**Heling Zhu**: Writing—original draft; writing—review & editing; conceptualization; methodology. **Tingting Dong**: Methodology; validation. **Tingtian Pang**: Validation; investigation. **Qiu Guan**: Validation; investigation. **Jingru Yang**: Methodology; validation. **Feini Zhao**: Writing—review & editing. **Bo Yang**: Writing—review & editing; Supervision. **Jie Qiu**: Supervision; funding acquisition.

## CONFLICT OF INTEREST STATEMENT

The authors declare no conflicts of interest.

## Data Availability

The supporting data in this study are available from the corresponding author on reasonable request.
